# Anterior Cingulate Cortex Contributes to Alcohol Withdrawal- Induced and Socially Transferred Hyperalgesia

**DOI:** 10.1523/ENEURO.0087-17.2017

**Published:** 2017-07-25

**Authors:** Monique L. Smith, Andre. T. Walcott, Mary M. Heinricher, Andrey E. Ryabinin

**Affiliations:** 1 Department of Behavioral Neuroscience, Oregon Health & Science University, Portland, OR 97239; 2 Department of Neurological Surgery, Oregon Health & Science University, OR 97239

**Keywords:** alcohol, anterior cingulate, empathy, pain, social, withdrawal

## Abstract

Pain is often described as a “biopsychosocial” process, yet social influences on pain and underlying neural mechanisms are only now receiving significant experimental attention. Expression of pain by one individual can be communicated to nearby individuals by auditory, visual, and olfactory cues. Conversely, the perception of another’s pain can lead to physiological and behavioral changes in the observer, which can include induction of hyperalgesia in “bystanders” exposed to “primary” conspecifics in which hyperalgesia has been induced directly. The current studies were designed to investigate the neural mechanisms responsible for the social transfer of hyperalgesia in bystander mice housed and tested with primary mice in which hyperalgesia was induced using withdrawal (WD) from voluntary alcohol consumption. Male C57BL/6J mice undergoing WD from a two-bottle choice voluntary alcohol-drinking procedure served as the primary mice. Mice housed in the same room served as bystanders. Naïve, water-drinking controls were housed in a separate room. Immunohistochemical mapping identified significantly enhanced Fos immunoreactivity (Fos-ir) in the anterior cingulate cortex (ACC) and insula (INS) of bystander mice compared to naïve controls, and in the dorsal medial hypothalamus (DMH) of primary mice. Chemogenetic inactivation of the ACC but not primary somatosensory cortex reversed the expression of hyperalgesia in both primary and bystander mice. These studies point to an overlapping neural substrate for expression of socially transferred hyperalgesia and that expressed during alcohol WD.

## Significance Statement

Pain is not a direct function of tissue damage, and is highly influenced by psychosocial context. Social influences on pain and underlying neural mechanisms have received limited attention in animal studies, although the available data suggest that social influences on pain in rodents are complex and bidirectional, as in humans. The present studies investigated mechanisms underlying hyperalgesia associated with alcohol withdrawal (WD), and with socially transferred hyperalgesia in “bystander” animals housed and tested in the same room, both of which could be considered top-down drivers of enhanced pain responding. Neural activity was differentially enhanced in the two groups, but chemogenetic inactivation pointed to an at least partially overlapping substrate for WD-related and socially transferred hyperalgesia in the anterior cingulate cortex (ACC).

## Introduction

The importance of social factors in the experience of pain has long been acknowledged in the clinical realm, where pain is often described as a “biopsychosocial” process (Gatchel et al., 2007; Lumley et al., 2011). Social influences on pain and underlying neural mechanisms have nonetheless received limited attention in animal studies, although the available data suggest that social influences on pain in rodents are complex and bidirectional, as more fully documented in humans (D'Amato and Pavone, 2012; Mogil, 2015). Social factors can depress or enhance pain in rodents. For example, social stress, such as repeated defeat by a dominant conspecific, results in antinociception in rodents (Miczek et al., 1985; Rodgers and Randall, 1986). Stress-related social odors have similarly been shown to produce antinociception in conspecifics (Fanselow, 1985). Social stress-induced analgesia is in part mediated by endogenous opioid systems, showing strong mechanistic parallels with other forms of stress-induced analgesia (Bodnar, 1986; Butler and Finn, 2009).

Social influences can also enhance pain. Cues emitted by individuals experiencing pain can elicit enhanced responsiveness in conspecifics. Thus, the ability to see acute pain behavior in a partner can lead to hyperalgesia, particularly if the partner is a cage mate (Langford et al., 2006; Li et al., 2014), and simply returning an animal to a cage following nociceptive testing results in shorter response latencies in animals subsequently tested from that same cage (Chesler et al., 2002). Naïve animals housed with conspecifics experiencing ongoing or periodic bouts of hyperalgesia exhibit socially transferred hyperalgesia (Devor et al., 2007; Baptista-de-Souza et al., 2015; Smith et al., 2016). For example, animals undergoing periodic (weekly) withdrawal (WD) from voluntary alcohol drinking display hyperalgesia, and exposure to olfactory cues from these withdrawn animals leads to hyperalgesia in otherwise naïve “bystander” mice housed and tested in the same room (Smith et al., 2016).

The experience of pain evoked by noxious stimulation reflects activity across a neural network that relays nociceptive information from the spinal cord to the cerebral cortex through cooperating ascending and descending pathways (Price, 2000; Rainville, 2002). In humans, a distributed cortical network including the primary and secondary somatosensory cortex, insula (INS), and anterior cingulate cortex (ACC) is reliably activated in association with pain (Price, 2000; Rainville, 2002; Apkarian et al., 2013a). Different structures within this network are thought to contribute preferentially to different aspects of the pain experience. The sensory-discriminative aspect of pain is attributed to recruitment of lateral parietal cortex, including primary somatosensory cortex (SI), whereas the affective element reflects activation of ACC via more medial transmission pathways. There is no clear agreement as to a specific role or roles of the INS in different aspects of pain, but it has been suggested to contribute to pain as a homeostatic emotion (Craig, 2003a,b).

There is evidence from functional imaging studies in humans that common cortical regions are engaged in pain evoked directly by noxious stimulation and by empathy for pain that is observed in others (Lamm et al., 2011). However, it is not clear that social modulation of pain in animals necessarily represents “empathy,” and mechanisms contributing to social transfer of pain and hyperalgesia in different social and behavioral contexts remain almost unexplored. The aim of the present study was to begin to elucidate the neural circuitry responsible for expression of socially transferred hyperalgesia.

We used a paradigm in which “primary” animals exhibited hyperalgesia during weekly periods of WD from voluntary alcohol drinking. Alcohol-naïve bystander mice were housed and tested in the same room, and developed comparable hyperalgesia (Smith et al., 2016). To compare circuitry mediating socially transferred hyperalgesia with that underlying the primary (alcohol WD induced) hyperalgesia, we analyzed Fos immunoreactivity (Fos-ir) in areas implicated in pain and empathy for pain in both primary and bystander mice. We also tested the effect of chemogenetic inactivation of the anterior cingulate and somatosensory cortices during the expression of WD-related and socially transferred hyperalgesia.

## Materials and Methods

Adult male C57BL/6J mice (total *n* = 95) from The Jackson Laboratory (https://www.jax.org/strain/000664) were used in these experiments. All mice were delivered at seven to eight weeks of age. On arrival, the mice were housed three to five per cage and spent at least one week acclimating to our colony room (12/12 h light/dark cycle; lights on 6 A.M.) before being subjected to any experimentation or stereotaxic surgery. For all experiments, mice were housed on a 12/12 h reverse light/dark cycle in a temperature (20-22°C)- and humidity-controlled environment with *ad libitum* access to food (LabDiet 5001; LabDiet) and tap water. All protocols were approved by the Oregon Health & Science University animal care and use committee and performed within the National Institutes for Health Guidelines for the Care and Use of Laboratory Animals, as well as the Guidelines for the Care and Use of Mammals in Neuroscience and Behavioral Research.

### Chemogenetic inhibition of circumscribed brain regions

Adeno-associated (serotype 8) inhibitory (hM4Di) Designer Receptors Exclusively Activated by Designer Drugs (DREADD) (AAV8-hSyn-hM3D/hM4D-Gi)-mCherry) virus (UNC Vector Core) was microinjected into ACC and SI as described below.

### Drugs

Clozapine-N-oxide (CNO; 1.0 mg/kg, Sigma) was dissolved in 0.5-1.0% dimethylsulfide (DMSO). Vehicle (Veh) consisted of saline with a matching percentage of DMSO. CNO and Veh were delivered intraperitoneally.

### Vector microinjections

One to two weeks before the start of each experiment, mice to be used in the DREADD experiments were transported to a suite for stereotactic surgery. Mice were anesthetized via 5% isoflurane delivered in oxygen. Following induction, mice were maintained under 1-2% isoflurane and secured in a stereotaxic frame (Kopf, 1900 series). A glass injector (0.5 mm OD) attached to a Hamilton syringe (1.0 μl) via plastic tubing was used to inject 150-300 nl (unilateral and bilateral, respectively) of vector into ACC (defined as CG1 in Paxinos and Franklin, 2004; unilateral, 40° medial angle, Anterior/posterior (A/P): +1.1 mm from Bregma, medial/lateral (M/L): 0.629 mm from midline, dorsal/ventral (D/V): 0.979 mm from the surface of the brain) and SI bilaterally (A/P: +0.98 mm from bregma, M/L ±3.1 mm, D/V: 1.375 mm). Unilateral injections were conducted within the ACC, due to the known connectivity between the right and left hemispheres, and because pilot studies indicated that unilateral inhibition was sufficient to block alcohol WD-induced hyperalgesia. All injections were accomplished over the course of 5 min, and injectors were left in place for 10 min and extracted over the course of 5 min to minimize tracking dorsally along the cannula track. Following recovery from anesthesia, mice were transported back to the animal colony and individually housed for 7-14 d to allow transfection of the virus and recovery from surgery. All mice then underwent the experimental procedures described below for alcohol drinking and mechanical testing.

#### DREADD activation

For all experiments, CNO (intraperitoneal) administration occurred immediately before placement on the testing apparatus on the final test session. Following a 20- to 30-min acclimation to the test rack and to allow for CNO distribution, mice were tested for mechanical sensitivity as described below. Immediately following the mechanical test, mice were killed via CO_2_ inhalation, and brains were extracted for placement analysis.

### Mechanical sensitivity

Responses (WD, shaking, or licking the paw) to mechanical stimulation of the plantar surface of the left hindpaw were determined with von Frey hairs (0.01-2 g plastic fibers) as an index of mechanical sensitivity. WD, shaking, or licking the paw was considered as a response. Mechanical thresholds were determined using the up-down technique (Chaplan, 1994). This method uses stimulus oscillation around the response threshold to determine the median 50% threshold of response. Mice were allowed to acclimate to the Plexiglas enclosure on top of a wire testing rack for 40 min on 2 d before the start of the experiment, and for 10-30 min before each test session. The testing rack was located within each testing room near the housing rack and illuminated with a dim red lamp. Mechanical sensitivity was assessed before treatment exposure (baseline), and mice were then assigned to treatment groups based on basal mechanical thresholds. Testing then occurred each week following 24 h of WD. A single experimenter conducted all behavioral testing. During testing, the experimenter was blind to the individual treatment assignments within each room.

### Alcohol intake procedures

Mice received 24-h access to two bottles with metal sipper tubes (containing water) on either side of the cage, with food evenly distributed along the wire cage top. No filter tops were used. Cages sizes were 7.25-inch W × 11.5-inch D × 5-inch H. After acclimation to the housing rooms, and baseline mechanical testing, mice either received access to two bottles of water only (bystanders and controls) or one bottle each water and alcohol (primary mice).

#### Twenty-four-hour access two-bottle choice

Primary mice received 24-h access to two bottles: one containing tap water and one containing increasing concentrations of ethanol (EtOH; 3-10%) dissolved in tap water. Fluid levels from each of the two bottles were recorded on a daily basis 2 h into the dark cycle. The locations of the bottles on the cages (left vs right) were alternated every other day to avoid the potential confound of an inherent side preference. Further, when multiple treatment groups were housed in a single room, the treatment assignment was randomly assigned across the cage locations, to avoid any confound related to the treatment of neighboring cages.

#### Alcohol WD

Once weekly (2 h into the dark cycle) EtOH bottles were removed and replaced with bottles containing water for 24 h. For the first week of drinking primary mice received 3% for 2 d, 6% EtOH for 2 d, and 10% EtOH for one day followed by 24 h of WD. In the second week, the mice were allowed access to 10% EtOH for 6 d followed by 24 h of WD.

### Tissue processing and immunohistochemistry

#### Fos

Mice (*n* = 27) from the behavioral experiment were used to examine Fos-ir. In these mice, brains were taken immediately after the second test session. Fos-ir would therefore correspond to the neural activation related to the state immediately before testing, which required 60-90 min. Mice were killed by CO_2_ inhalation; brains were extracted, postfixed for 24 h in 2% paraformaldehyde/PBS and cryopreserved using 20% and then 30% sucrose/PBS. Brains were sectioned at 30 μm. Sections containing 20 brain regions of interest were selected for analysis. Brain regions were defined using the Paxinos and Franklin (2001) Mouse Brain Atlas parameters. Some slices were damaged could not be analyzed, leading to inconsistent group sizes in some cases, but there were four to eight slices from at least five mice in each group used for analysis. The tissue was processed for Fos immunohistochemistry using standard avidin-biotin-DAB protocols (Ryabinin et al., 2000; Bachtell et al., 2002). Immunopositive cells were counted manually when there were relatively low numbers of cells, allowing for reliable counts. Thus, manual counts were conducted in all subcortical regions. For cortical regions where large numbers of cells exhibited Fos-ir, automatic cell counting was done using ImageJ (RRID:SCR_003070). An experimenter that was blind to treatment condition conducted all analyses. The immunohistochemical reaction was run twice, and the counts were averaged between the two sessions, with two to four slices for each region per mouse per batch (average of four to eight slices per mouse). This average served as a single data point for statistical analysis. There were no interactions of batch and factors of interest when batch was included in an ANOVA as a factor.

#### DREADD tissue processing

Following extraction, brains were postfixed for 24 h in 2% paraformaldehyde/PBS and cryopreserved in 20-30% sucrose/PBS. Brains were sliced at 30 μm and processed for mCherry and, in some cases, Fos immunohistochemistry. Unless noted otherwise, all steps were performed in 0.3% Triton-X/Tris-buffered saline (TBS) and preceded by three washes in TBS. The sections were rinsed for 30 min in 1% sodium borohydride in TBS, and blocked in 5% normal donkey serum (The Jackson Laboratory) for 45 min. The tissue was then incubated with 1:1000 goat polyclonal Fos antibody (Santa Cruz Biotechnology catalog number sc-52-G lot number RRID:AB_2629503) and 1:2500 rabbit polyclonal DS-Red (Clontech Laboratories catalog number 632496 lot number RRID:AB_10013483). This was followed by 1-h incubations with Alexa Fluor 555-labeled (Invitrogen catalog number A-31572 also A31572 lot number RRID:AB_162543) and Alexa Fluor 488-labeled secondary antibodies (raised in donkey, Invitrogen catalog number A-11055 also A11055 lot number RRID:AB_142672). Finally, slices were washed with PBS, mounted on gelatinized slides and coverslipped with Prolong Gold (Invitrogen). Colocalization of immunoreactivity was quantified manually using a Leica DM4000 microscope. Viral infusions were considered a “hit” when neuronal expression of the virus was limited to the boundaries of the chosen brain region (as defined by Paxinos and Franklin, 2001). When spread of the virus was beyond the target, data were not included. This procedure led to the following exclusions: 14 were excluded of 50 total ACC surgeries (final *n* = 36), and 3 exclusions of 26 of somatosensory cortex surgeries (final *n* = 23). Expression was seen from ∼0.43 to 0.62 rostral to bregma.

### Statistical analysis

For comparisons of mechanical sensitivity, dependent variables were analyzed by repeated measures ANOVA design with group (primary, bystander, control) and if applicable, treatment (Veh, CNO), as the between-subjects factors and mechanical test session (week/WD session) as the repeated measure. All immunohistochemistry results were analyzed via one-way ANOVA comparing between groups (primary, bystander, control). Significant interactions were followed by contrast analyses evaluating the impact of treatment and group on mechanical thresholds (Fishers LSD). For all analyses, significance threshold was set at *p* < 0.05. Data are expressed as mean ± SEM. All statistical analyses were performed with GraphPad Software Prism 6 (GraphPad Prism, RRID:SCR_002798) and are described in Table 1.

**Table 1.** Statistical analyses

	Data structure	Type of test	CI		Data structure	Type of test	CI
Table 2, row 1; Fig. 1C	Automated quantification of Fos+ cells in CG1 from two separate reactions. Counts were averaged between the 2 runs, with 2-4 slices for each region per mouse per batch (average of 4-8 slices per mouse)	One-way ANOVA with significant main effect of group followed by Fishers LSD *n* = 5-7 *p < 0.05	CTRL: (119.9-193.3) primary: (147.1-294.4) bystander: (195.9-308.0)	Table 2, row 14	Manual quantification of Fos+ cells in posteromedial cortical amygdaloid from two separate reactions. Counts were averaged between the 2 runs, with 2-4 slices for each region per mouse per batch (average of 4-8 slices per mouse)	One-way ANOVA *n* = 5-6 *p* > 0.05	CTRL: (5.040-14.10) primary: (4.533-10.61) bystander: (7.170-10.27)
Table 2, row 2	Automated quantification of Fos+ cells in CG2 from two separate reactions. Counts were averaged between the 2 runs, with 2-4 slices for each region per mouse per batch (average of 4-8 slices per mouse)	One-way ANOVA *n* = 5-7 *p* > 0.05	CTRL: (62.64-86.59) primary: (23.61-126.7) bystander: 41.03-113.6)	Table 2, row 15	Manual quantification of Fos+ cells in basolateral amygdala from two separate reactions. Counts were averaged between the 2 runs, with 2-4 slices for each region per mouse per batch (average of 4-8 slices per mouse)	One-way ANOVA *n* = 5-6 *p* > 0.05	CTRL: (6.156-19.07) primary: (5.469-18.79) bystander: (5.186-30.38)
Table 2, row 3	Automated quantification of Fos+ cells in GI from two separate reactions. Counts were averaged between the 2 runs, with 2-4 slices for each region per mouse per batch (average of 4-8 slices per mouse)	One-way ANOVA *n* = 5-7 p > 0.05	CTRL: (12.35-25.82) primary: (16.69-30.81) bystander: (8.518-40.40)	Table 2, row 16	Manual quantification of Fos+ cells in central nucleus of the amygdala from two separate reactions. Counts were averaged between the 2 runs, with 2-4 slices for each region per mouse per batch (average of 4-8 slices per mouse)	One-way ANOVA *n* = 5-6 *p* > 0.05	CTRL: (5.992-12.13) primary: (2.869-8.797) bystander: (3.828-16.21)
Table 2, row 4; Fig. 1D	Automated quantification of Fos+ cells in INS from two separate reactions. Counts were averaged between the 2 runs, with 2-4 slices for each region per mouse per batch (average of 4-8 slices per mouse)	One-way ANOVA with significant main effect of group followed by Fishers LSD *n* = 5-6 *p < 0.05	CTRL: (25.97-45.22) primary: (27.64-74.08) bystander: (29.72-116.3)	Table 2, row 17	Manual quantification of Fos+ cells in paraventricular nucleus from two separate reactions. Counts were averaged between the 2 runs, with 2-4 slices for each region per mouse per batch (average of 4-8 slices per mouse)	One-way ANOVA *n* = 5-6 *p* > 0.05	CTRL: (1.755-8.645) primary: (7.872-12.30) bystander: (0.972-25.83)
Table 2, row 5	Automated quantification of Fos+ cells in S1 from two separate reactions. Counts were averaged between the 2 runs, with 2-4 slices for each region per mouse per batch (average of 4-8 slices per mouse)	One-way ANOVA *n* = 5-6 *p* > 0.05	CTRL: (432.5-651.8) primary: (191.4-1128) bystander: (262.4-636.9)	Table 2, row 18	Quantification of Fos+ cells in the DMH from two separate reactions. Counts were averaged between the 2 runs, with 2-4 slices for each region per mouse per batch (average of 4-8 slices per mouse)	One-way ANOVA with significant main effect followed by Fishers LSD *n* = 6-7 **p = 0.007	CTRL: (10.64-21.66) primary: (17.37-43.81) bystander: (15.25-26.22)
Table 2, row 6	Manual quantification of Fos+ cells in dorsal lateral septum from two separate reactions. Counts were averaged between the 2 runs, with 2-4 slices for each region per mouse per batch (average of 4-8 slices per mouse)	One-way ANOVA *n* = 5-6 *p* > 0.05	CTRL: (3.120-10.67) primary: (1.763-12.15) bystander: (3.195-9.805)	Table 2, row 19	Manual quantification of Fos+ cells in the centrally projecting Edinger Westphal from two separate reactions. Counts were averaged between the 2 runs, with 2-4 slices for each region per mouse per batch (average of 4-8 slices per mouse)	One-way ANOVA *n* = 5-6 *p* > 0.05	CTRL: (4.866-11.11) primary: (4.261-20.06) bystander: (6.974-15.78)
Table 2, row 7	Manual quantification of Fos+ cells in intermediate lateral septum from two separate reactions. Counts were averaged between the 2 runs, with 2-4 slices for each region per mouse per batch (average of 4-8 slices per mouse)	One-way ANOVA *n* = 5-6 *p* > 0.05	CTRL: (18.13-29.51) primary: (12.99-25.89) bystander: (20.04-27.38)	Table 2, row 20	Manual quantification of Fos+ cells in periaqueductal gray from two separate reactions. Counts were averaged between the 2 runs, with 2-4 slices for each region per mouse per batch (average of 4-8 slices per mouse)	One-way ANOVA *n* = 5-6 *p* > 0.05	CTRL: (27.73-62.22) primary: (30.75-129.2) bystander: (42.31-85.50)
Table 2, row 8	Manual quantification of Fos+ cells in ventral lateral septum from two separate reactions. Counts were averaged between the 2 runs, with 2-4 slices for each region per mouse per batch (average of 4-8 slices per mouse)	One-way ANOVA *n* = 5-6 *p* > 0.05	CTRL: (11.23-16.07) primary: (5.770-22.95) bystander: (5.559-18.98)	Table 2, row 21	Manual quantification of Fos+ cells in substantia nigra from two separate reactions. Counts were averaged between the 2 runs, with 2-4 slices for each region per mouse per batch (average of 4-8 slices per mouse)	One-way ANOVA *n* = 5-6 *p* > 0.05	CTRL: (-0.945-10.30) primary: (1.372-14.46) bystander: (2.184-10.03)
Table 2, row 9	Manual quantification of Fos+ cells in nucleus accumbens from two separate reactions. Counts were averaged between the 2 runs, with 2-4 slices for each region per mouse per batch (average of 4-8 slices per mouse)	One-way ANOVA *n* = 5-6 *p* > 0.05	CTRL: (5.400-37.91) primary: (10.44-41.08) bystander: (15.03-31.01)	Table 2, row 22	Manual quantification of Fos+ cells in ventral tegmental Area from two separate reactions. Counts were averaged between the 2 runs, with 2-4 slices for each region per mouse per batch (average of 4-8 slices per mouse)	One-way ANOVA *n* = 5-6 *p* > 0.05	CTRL: (-1.562-8.729) primary: (-4.473-20.07) bystander: (-9.419-31.42)
Table 2, row 10	Manual quantification of Fos+ cells in anterior bed nucleus of the stria terminalis from two separate reactions. Counts were averaged between the 2 runs, with 2-4 slices for each region per mouse per batch (average of 4-8 slices per mouse)	One-way ANOVA *n* = 5-6 *p* > 0.05	CTRL: (25.51-36.28) primary: (19.48-76.52) bystander: (25.76-52.53)	Fig. 1B	Comparison of mechanical thresholds of each group over 3 test sessions	Two-way RM ANOVA, significant for test session *n* = 6-7 *p < 0.05	CTRL: (1.521-2.143) primary: (-0.255-2.787) bystander: (0.031-2.950)
Table 2, row 11	Manual quantification of Fos+ cells in posterior bed nucleus of the stria terminalis from two separate reactions. Counts were averaged between the 2 runs, with 2-4 slices for each region per mouse per batch (average of 4-8 slices per mouse)	One-way ANOVA *n* = 5-6 *p* > 0.05	CTRL: (-1.066-17.96) primary: (0.4374-5.382) bystander: (1.829-7.948)	Fig. 2F	Comparison of mechanical thresholds of each group following treatment with Veh or CNO on the second test session (inhibition of the ACC)	Two-way ANOVA, significant main effect of treatment and significant interaction followed by Fishers LSD *n* = 5-7 *p < 0.05	CTRL/Veh: (0.76-1.68) CTRL/CNO: (0.70-1.54) bystander/VEH: (0.31-0.76) bystander/CNO: (0.39-1.85) primary/Veh: (0.16-0.65) primary/CNO:(0.36-2.14)
Table 2, row 12	Manual quantification of Fos+ cells in dentate gyrus from two separate reactions. Counts were averaged between the 2 runs, with 2-4 slices for each region per mouse per batch (average of 4-8 slices per mouse)	One-way ANOVA *n* = 5-6 *p* > 0.05	CTRL: (24.09-29.40) primary: (22.46-55.44) bystander: (28.60-38.16)	Fig. 2G	Comparison of mechanical thresholds of each group following treatment with Veh or CNO on the second test session (inhibition of S1)	Two-way ANOVA, significant main effect of group, no interaction *n* = 5-7 *p < 0.0001	CTRL/Veh: (-7.73-11.46) CTRL/CNO: (0.368-2.32) bystander/VEH: (-0.088-1.23) bystander/CNO: (-4.63-6.18) primary/Veh: (-0.62-1.91) primary/CNO:(-0.80-3.36)
Table 2, row 13	Manual quantification of Fos+ cells in posterolateral cortical amygdaloid from two separate reactions. Counts were averaged between the 2 runs, with 2-4 slices for each region per mouse per batch (average of 4-8 slices per mouse)	One-way ANOVA *n* = 5-6 *p* > 0.05	CTRL: (18.99-30.39) primary: (8.949-25.65) bystander: (13.75-19.45)				

## Results

### Differential Fos activation in bystander and primary mice

**Figure 1.**
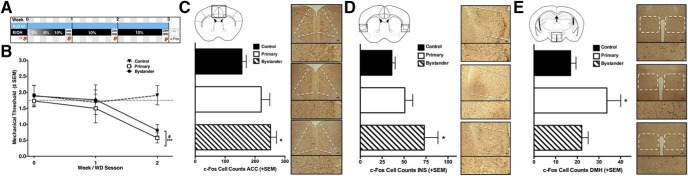
Differentially enhanced Fos in primary and bystander mice. ***A***, Timeline of data collection. Blue bar represents bystander and control mice, black bar represents primary mice, with corresponding EtOH concentration at different times (% v/v); VF and orange arrows represent von Frey testing at the end of the WD period. ***B***, Primary (*n* = 6) and bystander (*n* = 6) mice demonstrate significant decreases in mechanical thresholds compared to separately housed controls (*n* = 7; *F*_(2,16)_ = 9.68, *p* = 0.002). Fos-positive cell counts in three brain regions revealed significant differences among groups, the ACC (***C***; bregma 1.1-0.5), INS (***D***; bregma 1.1-0.5), and the DMH (***E***; bregma -1.4 to -0.94). Brain regions are shown schematically in the top left of each panel, with representative photomicrographs of each treatment group in corresponding order on the right (bystander, black bar/top right; primary, white bar/middle right; control, striped bar/bottom right; scale bar, 100 μm). Bystander mice displayed an increase in the number of c-Fos cells in the ACC (*F*_(2,14)_ = 4.8, *p =* 0.026), and in INS (*F*_(2,15)_ = 3.8, *p =* 0.046) compared to the controls. By contrast, the primary group displayed enhanced Fos-ir in the DMH (*F*_(2,16)_ = 4.8, *p* = 0.007). *p < 0.05 compared to the control group based on Fishers LSD.

Primary mice were allowed constant voluntary access to alcohol for 6 d followed by 24-h sessions of WD, for a total of two weeks (two WD sessions a week apart; Fig. 1*A*). Mechanical sensitivity was tested at the end of each WD session using calibrated von Frey filaments to measure the threshold for removal of the paw from the stimulus. These primary mice demonstrated significant hypersensitivity during WD, and this hyperalgesia was transferred to bystander mice that were housed and tested in the same room at the same time, but drinking only water (Fig. 1*B*).

To explore the neural circuits involved in the expression of hyperalgesia manifest by both primary and bystander mice, we compared brain Fos-ir of these groups compared to water-drinking control mice housed and tested in a separate room. We examined Fos as a measure of neural activation during the second of the two weekly sessions of abstinence from alcohol (WD), as brains were taken immediately following the completion of the second, final mechanical test. Between-group differences were seen in three of 21 brain regions analyzed Table 2. When compared to the control group, bystander mice demonstrated enhanced Fos-ir in the CG1 subregion of the ACC (Fig. 1*C*) and the INS (Fig. 1*D*). By contrast, increased Fos-ir was seen in the dorsal medial hypothalamus (DMH) in primary mice, but not bystander mice, compared to controls (Fig. 1*E*).

**Table 2.** Fos-ir in primary and bystander mice

Brain area	Control	Primary	Bystander	ANOVA
**Anterior cingulate (CG1)**	156.6 ± 14.28	220.8 ± 28.66	251.9 ± 20.18*	***F*_(2,14)_ = 4.77**
Anterior cingulate (CG2)	73.56 ± 5.65	75.14 ± 20.05	77.33 ± 14.12	*F*_(2,15)_ = 0.017
GranularInsula (GI)	19.08 ± 2.752	23.89 ± 2.695	24.46 ± 6.201	*F*_(2,16)_ = 0.547
**Agranular insula (INS)**	36.1 ± 3.73	50.86 ± 9.032	73.02 ± 15.59*	***F*_(2,15)_ = 3.779**
Somatosensory	542.1 ± 44.79	659.8 ± 168.7	449.6 ± 76.52	*F*_(2,16)_ = 1.147
Dorsal lateral septum	6.893 ± 1.542	6.958 ± 2.021	6.5 ± 1.351	*F*_(2,17)_ = 0.023
Intermediate lateral septum	23.82 ± 2.326	23.71 ± 1.429	19.44 ± 2.637	*F*_(2,17)_ = 1.250
Ventral lateral septum	13.65 ± 0.991	14.36 ± 3.342	12.27 ± 2.743	*F*_(2,17)_ = 0.183
Nucleus accumbens	21.65 ± 6.323	25.76 ± 5.959	23.02 ± 3.265	*F*_(2,16)_ = 0.155
Bed nucleus of the stria terminalis (anterior)	30.89 ± 2.2	48 ± 11.1	39.14 ± 5.47	*F*_(2,17)_ = 1.561
Bed nucleus of the stria terminalis (posterior)	20.53 ± 8.444	25.45 ± 2.91	23.33 ± 4.889	*F*_(2,15)_ = 1.472
Dentate gyrus	36.19 ± 5.337	39.14 ± 6.744	31.27 ± 6.609	*F*_(2,17)_ = 0.4011
Posterolateral cortical amygdaloid	24.69 ± 2.328	17.3 ± 3.412	16.6 ± 1.027	*F*_(2,16)_ = 2.845
Posteromedial cortical amygdaloid	9.571 ± 1.852	7.571 ± 1.242	8.722 ± 0.604	*F*_(2,17)_ = 0.5452
Basolateral amygdala	12.61 ± 12.61	12.13 ± 2.592	17.78 ± 4.901	*F*_(2,15)_ = 0.7956
Central nucleus of the amygdala	9.063 ± 1.194	5.833 ± 1.153	10.02 ± 2.409	*F*_(2,15)_ = 1.687
Paraventricular nucleus	3.4 ± 0.75	11.17 ± 3.55	12.13 ± 3.29	*F*_(2,12)_ = 2.584
**Dorsal medial hypothalamus**	16.15 ± 2.251	30.59 ± 5.143**	20.73 ± 2.242	*F*_(2,16)_ = 4.842
Centrally projecting Edinger Westphal	7.988 ± 1.276	12.16 ± 3.073	11.38 ± 1.799	*F*_(2,17)_ = 1.150
Periaqueductal gray	44.98 ± 7.049	79.96 ± 19.14	63.9 ± 8.827	*F*_(2,17)_ = 2.073
Substantia nigra	4.679 ± 2.298	7.917 ± 2.546	6.107 ± 1.603	*F*_(2,17)_ = 0.5495
Ventral tegmental area	2.93 ± 1.02	4.88 ± 2.54	9.43 ± 4.66	*F*_(2,15)_ = 1.115

Mean (±SEM) c-Fos-positive cell counts for experimental each group per brain area examined. ANOVA values are presented in the right column, with significant main effects of group in bold. *p < 0.05 contrast to controls, Fishers LSD.

### Chemogenetic inhibition of the anterior cingulate, but not primary somatosensory cortex, reverses hyperalgesia in primary and bystander mice

**Figure 2.**
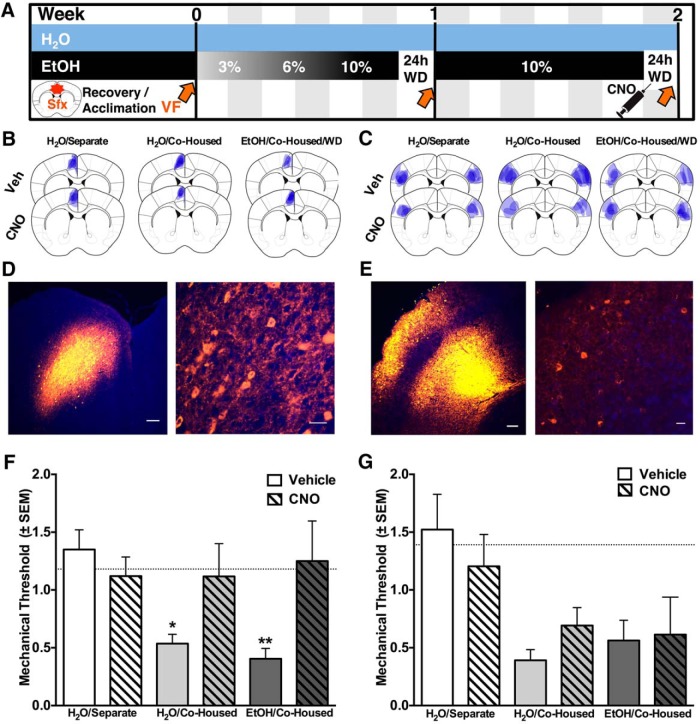
Inhibition of ACC, but not somatosensory cortex reverses hyperalgesia in primary and bystander mice. ***A***, Timeline of data collection and experimental manipulation: Sfx refers to surgery, which took place 7-14 d before beginning of experiments. Blue bar represents bystander and control mice, black bar represents primary mice, with corresponding EtOH % (v/v); VF and orange arrows represent von Frey testing at end of 24-h abstinence; WD represents withdrawal from alcohol; black syringe represents CNO injection (20 min before the second mechanical test). ***B***, ***C***, Overlapping pattern of viral expression (blue) for each treatment group in the ACC and SI. Representative photomicrographs of hM4Di viral expression, within the (***D***) ACC (orange) with DAPI in blue (***E***) SI (orange) and DAPI in blue. Left panels: scale bars, 100 μm; right panels: scale bars, 20 μm. ***F***, Animals in which DREADDs were expressed in ACC and given Veh (*n* = 5, primary; *n* = 5, bystander) showed a significant decrease in threshold compared to Veh-treated separately housed control animals (*n* = 8). By contrast, animals given CNO before testing (*n* = 6, primary; *n* = 6, bystander) showed no hyperalgesia compared to CNO-treated controls (*n* = 6). According to ANOVA, this led to a significant difference of treatment (*F*_(1,30)_ = 4.79, *p =* 0.037), as well as a significant interaction (*F*_(2,30)_ = 3.37, *p =* 0.048). CNO groups were no longer significantly different from separately housed controls according to Fishers LSD. ***G***, Bystander (*n* = 5-7/group) and primary (*n* = 6/group) mice bilaterally transfected with the hM4Di DREADD virus in SI demonstrated significant decreases in mechanical thresholds on the second WD session compared to separately housed controls (*n* = 5-6), leading to significant differences between groups (*F*_(2,29)_ = 14.88, *p <* 0.0001), but no significant effects of CNO treatment or an interaction, indicating that inactivation of SI had no effect on hypersensitivity. Mean basal responses of all groups represented by dotted line (—). *p < 0.05 compared to controls receiving the same treatment, according to Fishers LSD.

To determine if the ACC is required for the expression of mechanical hypersensitivity, we used DREADDs (Armbruster et al., 2007; Rogan and Roth, 2011), synthetic G-protein coupled receptors that display selective sensitivity to the pharmacologically inert drug CNO. At least one week before the start of the experiment (Fig. 2*A*) mice were transfected with an AAV vector carrying a Gi-coupled inhibitory DREADD (hM4Di) microinjected unilaterally into the ACC (Fig. 2*B*,*D*). Before the second WD session, mice were injected with either CNO or Veh. There was no difference between Veh-treated and CNO-treated controls (separately housed). Veh-treated bystander and primary animals showed significantly lower thresholds than Veh-treated controls. By contrast, the thresholds of CNO-treated bystander and primary animals were not significantly different from CNO-treated controls (Fig. 2*F*). When the same DREADD construct was transfected into SI (Fig. 2*C*,*E*) there was no effect of CNO in primary or bystander mice (Fig. 2*G*), compared to Veh injection. Again, there was no difference in mechanical thresholds of separately housed control mice transfected with the DREADD virus in the somatosensory cortex and treated with CNO or Veh (Fig. 2*G*). These results indicate that the ACC, but not SI, is required for the expression of both socially transferred and alcohol WD-induced hyperalgesia

## Discussion

These results confirm social transfer of alcohol WD-induced hyperalgesia from withdrawn primary mice to bystander mice housed in the same room, and begin to elucidate the neural mechanisms involved in the expression of these behaviors. Examination of Fos-ir revealed enhanced neural activity in the ACC and INS of bystander mice when compared to water-drinking controls housed in a separate room. Further, chemogenetic inactivation of the ACC showed that this area is required for the expression of mechanical hypersensitivity in these bystander mice.

Alcohol-withdrawn mice also demonstrated an enhancement in Fos-ir within the ACC that was comparable to that of bystanders, but this increase was not statistically different from separately housed control mice. Notably, both primary and bystander groups receive pain-related social cues from their neighbors, and this may contribute to the neural activation seen in the ACC. If the ACC is important to the perception and/or expression of socially induced hyperalgesia, it is perhaps unsurprising that inhibition of the ACC interferes with this behavior in both primary and bystander groups. Alternatively, these results could indicate that the ACC is integral to expression of alcohol WD-induced hyperalgesia specifically, but the design of the current studies did not allow us to test this possibility.

Notably, the c-Fos results point to several other brain regions that may be of importance to the alcohol WD-induced and/or socially transferred hyperalgesia. For both primary and bystander mice there were trends toward enhanced c-Fos activity in the paraventricular nucleus of the hypothalamus, periaqueductal gray, and centrally projecting Edinger Westphal nucleus, indicating a larger overlapping neural circuit than we explored in the current studies. Furthermore, there was a significant enhancement in Fos-ir within the DMH of only the primary mice, suggesting that this area is distinctly important for alcohol WD, but not engaged as part of socially transferred hyperalgesia. Conversely, significantly enhanced activity was identified in the INS of only the bystander mice, indicating a possible divergent neural circuit underlying socially induced hyperalgesia. The current studies create a foundation for future research to explore the necessity of each of the brain regions for alcohol WD-induced and socially transferred hyperalgesia.

Many commonly used models of chronic pain and hyperalgesia are triggered by a “bottom-up” process, typically characterized by localized inflammatory or neuropathic insult that leads to hyperalgesia centered around the insult. By contrast, the two forms of hyperalgesia studied here do not have a peripheral basis in tissue or nerve injury. Although prolonged exposure to alcohol can produce neuropathy, hyperalgesia in the short-term voluntary drinking paradigm is associated specifically with WD, and not alcohol-exposure per se, which rules out a peripheral neuropathy (Smith et al., 2016). In addition, both WD-associated and socially transferred hyperalgesia can be demonstrated using a range of stimulus modalities, including thermal and chemical stimuli, as well as the mechanical stimulation used here (Smith et al., 2016). It is thus likely that hyperalgesia in both groups was diffuse, and not restricted to the tested hindpaw. It is therefore reasonable to suggest that both WD-induced and socially transferred hyperalgesia represent a top-down process, with overlap in the ACC. This speculation is supported by previous literature that has demonstrated that the ACC can play a role in facilitating spinal nociceptive transmission (Calesejan et al., 2000; Koga et al., 2016).

ACC is not pain specific and is involved in general affect, attention and motor preparation (Devinsky et al., 1995). However, block of ACC has at most minor effects on acute nociceptive reflexes (Jeon et al., 2010; Bliss et al., 2016), which suggests that the effect of ACC inactivation was not a motor confound. Nevertheless, the involvement of the ACC could be related not to the hyperalgesia in these mice, but to some other aspect of the experience. Inactivation of ACC interferes with acquisition of fear of an environmental context in which a conspecific was seen to receive footshock (Jeon et al., 2010), and permanent lesions of ACC prevent acquisition of hyperalgesia in cage mates observing their partner’s response to acute irritant treatment (bee venom; Li et al., 2014). ACC could thus be seen as necessary for observational learning, whether fear or hyperalgesia was acquired. In the present experiments, the ACC was intact during acquisition of hyperalgesia, and was only blocked at the time of sensory testing, indicating that the ACC contributes to expression of socially transferred hyperalgesia. Finally, considering the multiple, functionally distinct cell populations within the ACC, it is possible that our inhibition studies targeted multiple cell populations with potentially varied involvement in socially transferred pain. Nevertheless, we were able to see a reversal of hypersensitivity in both primary and bystander animals, indicating the overall importance of the ACC to this behavior. In future studies, it would be interesting to determine if there are distinct circuits within the ACC governing alcohol WD-induced and socially transferred hyperalgesia.

It is possible that the lack of an effect of CNO in the control animals is due to a ceiling effect, and the von Frey testing was not able to detect analgesic effects at baseline. However, this seems unlikely, as treatment with analgesic drugs can indeed increase mechanical threshold at baseline in the mouse (e.g., Fuchs et al., 1999; Scherrer et al., 2009). Furthermore, our data are in accord with previous research that demonstrates that inactivation of the ACC has an antihyperalgesic effect, rather than an analgesic effect (LaGraize and Fuchs, 2007).

Bilateral inhibition of the somatosensory cortex did not alter hypersensitivity in either primary or bystander mice, highlighting the relative importance of the ACC in this behavior and serving as an important control for non-specific effects of CNO. This finding is in accord with studies showing that ablation of somatosensory cortex does not eliminate pain behaviors or experience (Bushnell et al., 1999; Treede et al., 1999). It is also consistent with the Fos-ir data in the present study, as activity was not enhanced within the somatosensory cortex of either group.

The Fos studies showed that the DMH is activated in primary, but not bystander, mice. This region is thought to coordinate autonomic sequelae to mild, or “emotional,” stress, including increases in heart rate, blood pressure and body temperature (DiMicco et al., 2002; Fontes et al., 2011; Dampney, 2015). DMH is also recruited in moderate stress paradigms to produce hyperalgesia via descending pronociceptive pathways projecting from the brainstem to the dorsal horn (Martenson et al., 2009; Wagner et al., 2013). However, hyperalgesia during alcohol WD is not reversed by inhibition of corticosteroid synthesis or accompanied by other behavioral indicators of intense stress (e.g., changes in behavior in the elevated plus maze; Smith et al., 2016). Moreover, intense stress has been shown repeatedly to lead to hypoalgesia in observers rather than hyperalgesia (Miczek et al., 1985; Rodgers and Randall, 1986; Kavaliers et al., 2001), and blocking glucocorticoid signaling in an observer to reduce social stress can unmask socially transferred hyperalgesia (Martin et al., 2015). Viewed collectively, these lines of evidence argue that while alcohol WD may involve some stress-related elements, exposure to social cues related to alcohol WD does not.

Pain is often reported as a symptom of alcohol WD in humans. The present findings support a relationship between alcohol abuse and pain disorders, and add to evidence that symptoms related to alcohol abstinence rely on neural circuitry that is fundamental to the experience of pain (Egli et al., 2012; Apkarian et al., 2013b).

In summary, these studies confirm the social transfer of hypersensitivity from mice experiencing alcohol WD to bystander mice housed and tested in the same room. They demonstrate that the ACC is necessary for expression of both forms of hyperalgesia, pointing to an overlapping neural substrate for expression of socially transferred hyperalgesia and that expressed during alcohol WD.

## Synthesis

Reviewing Editor: Karen Davis, Krembil Research Institute and University of Toronto Decisions are customarily a result of the Reviewing Editor and the peer reviewers coming together and discussing their recommendations until a consensus is reached. When revisions are invited, a fact-based synthesis statement explaining their decision and outlining what is needed to prepare a revision will be listed below. The following reviewer(s) agreed to reveal their identity: Min Zhuo.

This study, explored the roles of cortical regions in alcohol withdrawal induced, and socially transferred hyperalgesia.

The authors use a recently reported social transmission of pain model to investigate the underlying neural circuits that might mediate this process. They demonstrate that cfos activation primarily is observed in the bystander mice in ACC and INS, while Fos is induced in the DMH only in primary mice. They also reported that chemogenetic inhibition of the ACC reverses the hyperalgesia, but inhibiting the SSCX does not. ACC inhibition had no impact on controls, suggesting a more specific role of ACC in pain in the pain-exposed groups.

Overall then, the authors have identified the involvement of the ACC in this novel transferred hyperalgesia. Using optogenetic methods, they showed that inactivation of ACC is critical for this hyperalgesia. These results are new, and potentially important. While these results are interesting and suggestive, there are concerns and missing results that need to be addressed:

1. If possible, Fig 1C would benefit from results to indicate whether the trend for increased Fos in ACC in the primary mice is significant or not. If this data could be obtained it would resolve the observation that ACC inhibition reduces hyperalgesia in primary and bystander mice, while the Fos increase is at present significant only in bystanders. Also, there are regions (such as PV nucleus and PAG) where there are also clear trends but the sample size is likely underpowered. The authors are encouraged to try to add the data to address these concerns.

2. While the focus seems to be on “overlapping” regional control of hyperalgesia (through the ACC), there clearly are also differences between primary and bystander (especially DMH), and it would be important and interesting to identify the physiological importance, e.g. of inhibiting INS (which might impact only the bystander mice).

3. The INS is described as AI in the Table, while more posterior insula is shown in figure 1.

4. Is the lack of effect of CNO in controls a reflection of a ceiling effect on the mechanical threshold that can be measured?

5. The authors suggested that top-down modulation may be responsible for hyperalgesia. In fact, there are several previous publications indicate that ACC exerts descending facilitatory effects on spinal nociceptive transmission (see Calejesan et al., 2000; Chen et al., 2015; and for review Zhuo 2016). The authors should cite these works to support this speculation.

6. An alternative explanation for the transferred hyperalgesia is that there is a supraspinal mechanism without affecting spinal sensory transmission. It is possible that ACC neurons may facilitate the motor withdrawal pathways. This could be discussed.
